# Supplementary figures and images for: Gut commensal bacteria influence colorectal cancer development by modulating immune response in AOM/DSS-treated mice
Source: Microbiol Spectr. 2025 May 16;13(7):e02792-24. doi: 10.1128/spectrum.02792-24 (PMC12211034; doi:10.1128/spectrum.02792-24)

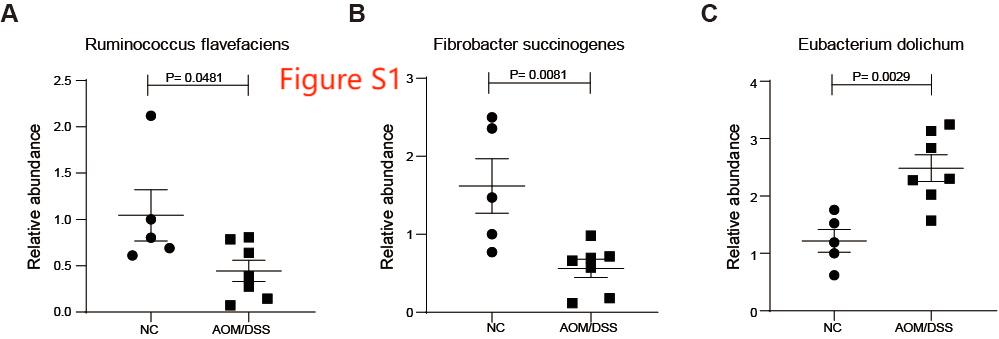

Supplement: Figure S1 — Relative abundance of (A) Ruminococcus flavefaciens, (B) Fibrobacter succinogenes, and (C) Eubacterium dolichum in fecal samples from mice. [file spectrum.02792-24-s0001.jpg]

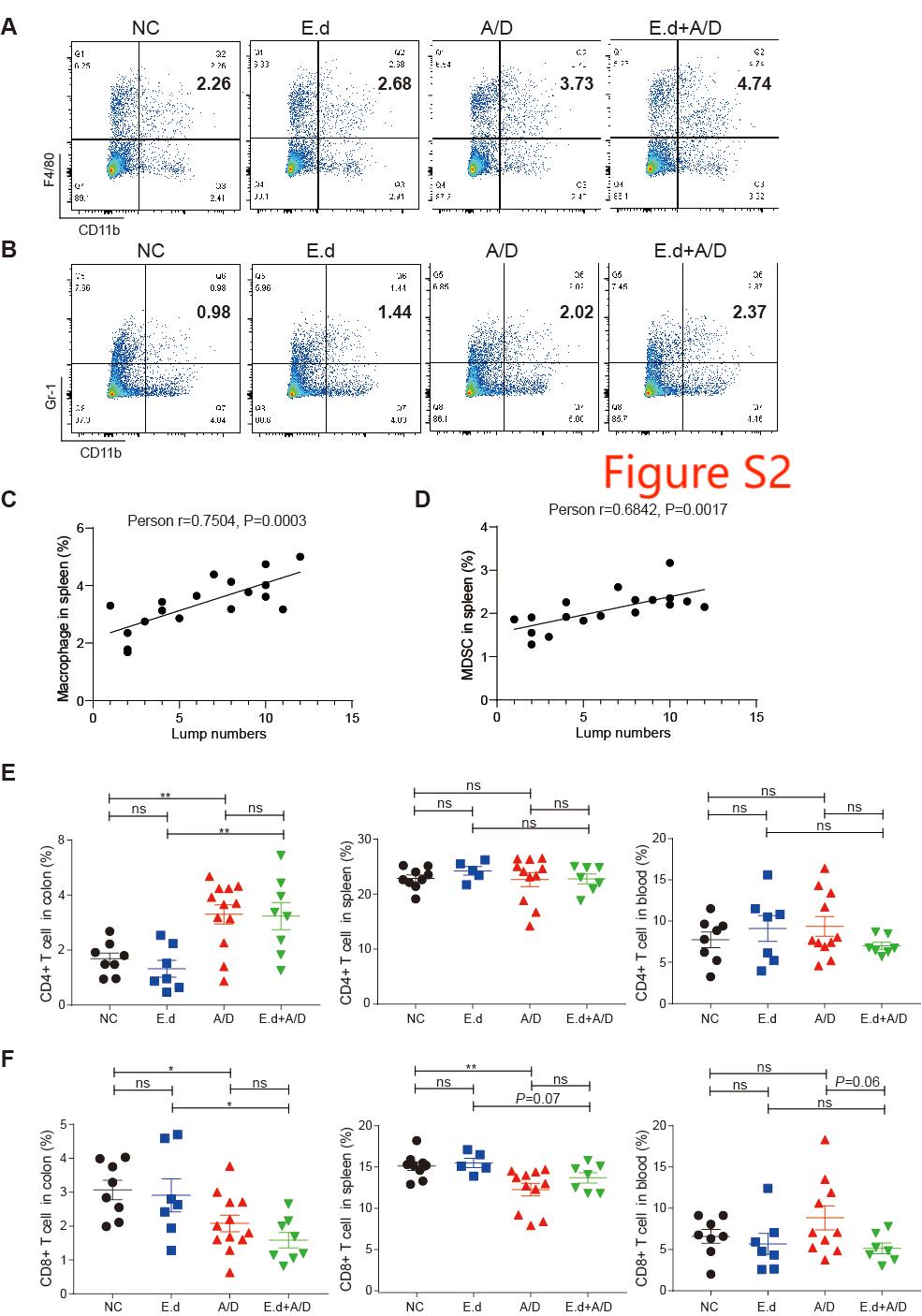

Supplement: Figure S2 — Relative abundances of immune cells in mice treated with AOM/DSS and 3 cycles of E.d. [file spectrum.02792-24-s0003.jpg]

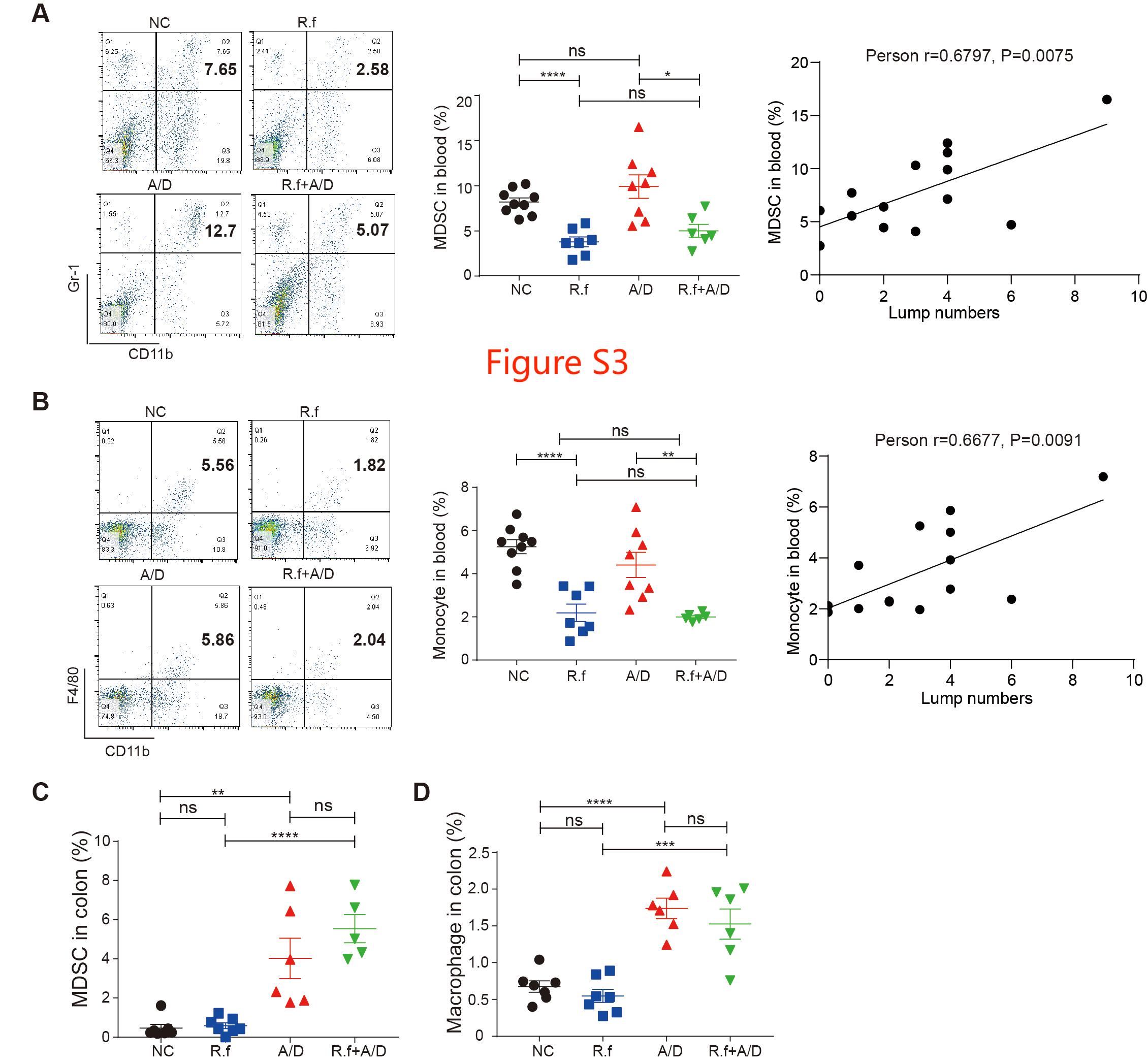

Supplement: Figure S3 — Relative abundances of immune cells in mice treated with AOM/DSS and 4 cycles of R.f. [file spectrum.02792-24-s0004.jpg]

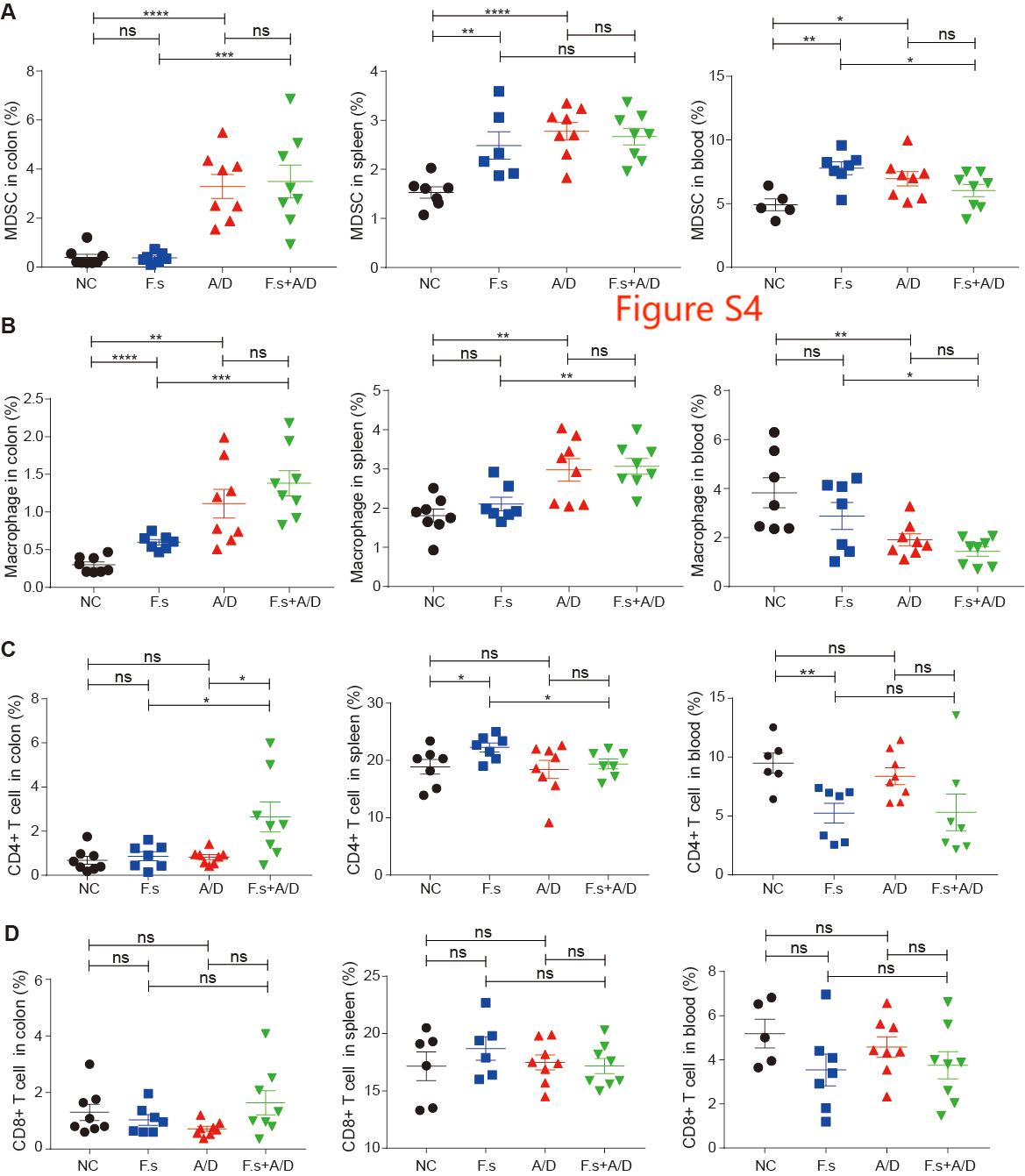

Supplement: Figure S4 — Relative abundances of immune cells in mice treated with AOM/DSS and 3 cycles of F.s. [file spectrum.02792-24-s0005.jpg]

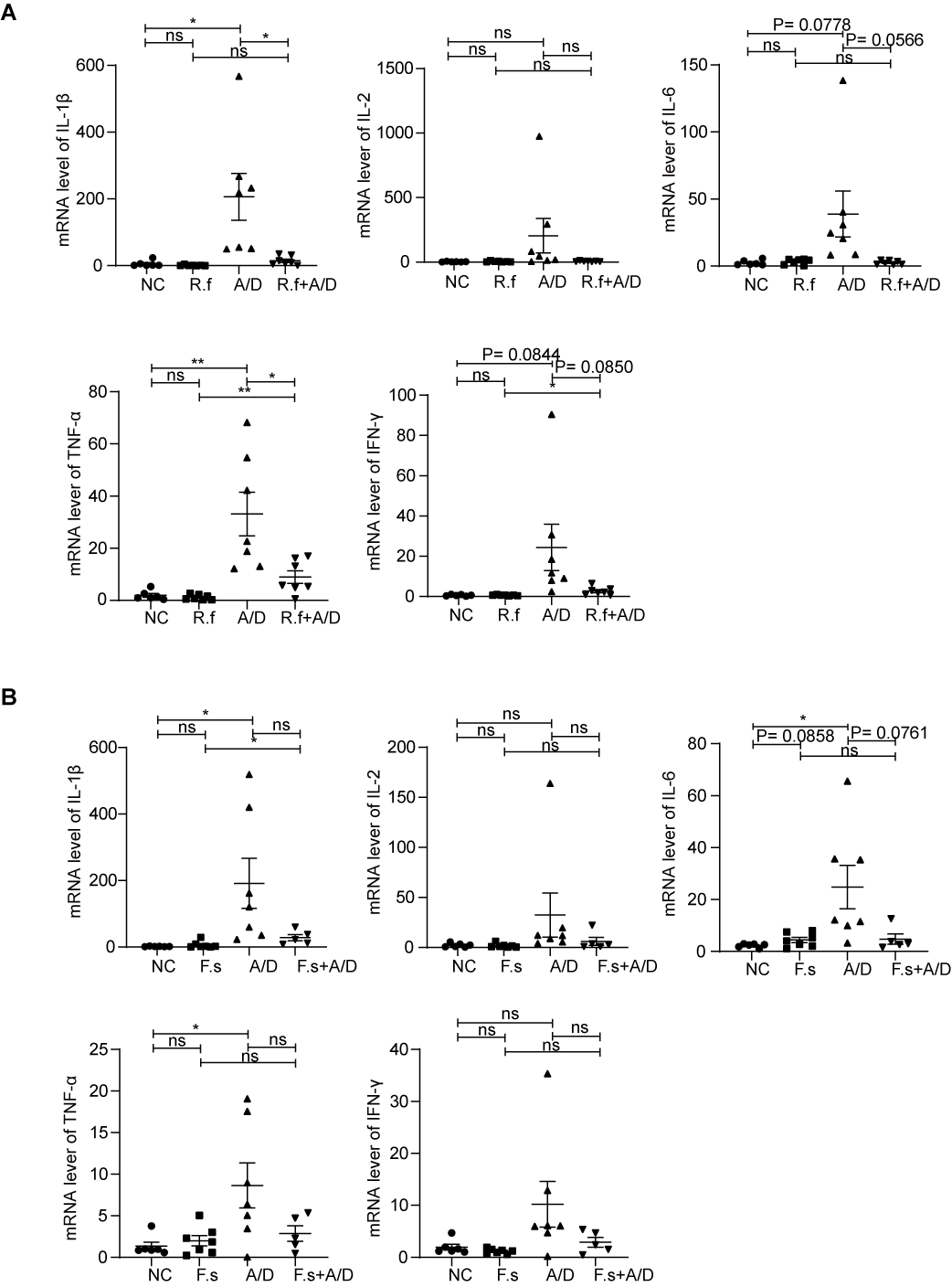

Supplement: Figure S5 — Relative abundances of pro-inflammatory markers in a mouse colon after AOM/DSS induction and R.f/F.s administration. [file spectrum.02792-24-s0006.png]
