# Supplementary material for: Gut commensal bacteria influence colorectal cancer development by modulating immune response in AOM/DSS-treated mice
Source: Microbiol Spectr. 2025 May 16;13(7):e02792-24. doi: 10.1128/spectrum.02792-24 (PMC12211034; doi:10.1128/spectrum.02792-24)
Supplement: Supplemental figure legends and tables — S1 to S3. [file spectrum.02792-24-s0002.docx]

**Figure legends for Supplementary Figures**

**Figure S1.** **Relative abundance of (A) *Ruminococcus flavefaciens*, (B) *Fibrobacter succinogenes,* and (C) *Eubacterium dolichum* in fecal samples from mice between NC group and AOM/DSS treated- group by qPCR**

Data are expressed as the mean ± SEM. NC group: n = 5, AOM/DSS group: n = 7.

**Figure S2. Relative abundances of immune cells in mice treated with AOM/DSS and 3 cycles of E.d**

**(A, B)** Representative FACS scatter diagrams of MDSCs defined by CD11b^+^ and Gr-1^+^ **(A)** and macrophages defined by CD11b^+^ and F4/80^+^ **(B)** at the end of the experiment in the spleens of mice. **(C, D)** Correlation analysis between tumor numbers and percentages of MDSCs **(C)** and macrophages **(D)**. **(E)** Relative abundances of CD4^+^ T cells defined by CD3^+^ and CD4^+^ in the colons, spleens, and blood of mice. **(F)** Relative abundances of CD8^+^ T cells defined by CD3^+^ and CD8^+^ in the colons, spleens, and blood of mice. Data are expressed as the mean ± SEM. **p <* 0.05; ***p <* 0.01.

**Figure S3. Relative abundances of immune cells in mice treated with AOM/DSS and 4 cycles of R.f**

**(A, B)** Relative abundances of MDSCs defined by CD11b^+^ and Gr-1^+^ **(A)** and macrophages defined by CD11b^+^ and F4/80^+^ **(B)** at the end of the experiment in the blood of mice (left panel: representative FACS scatter diagram; middle panel: FACS statistical results; right panel: correlation analysis between percentages of immune cells and tumor numbers). **(C,D)** Relative abundances of MDSCs **(C)** and macrophages **(D)** at the end of the experiment in the colons of mice. Data are expressed as the mean ± SEM. **p* < 0.05; ***p* < 0.01; ****p* < 0.001; *****p* < 0.0001.

**Figure S4. Relative abundances of immune cells in mice treated with AOM/DSS and 3 cycles of F.s**

**(A)** Relative abundances of MDSCs at the end of the experiment in the colons, spleens and blood of mice. **(B)** Relative abundances of macrophages at the end of the experiment in the colons, spleens and blood of mice **(C)** Relative abundances of CD4^+^ T cells at the end of the experiment in the colons, spleens and blood of mice. **(D)** Relative abundances of CD8^+^ T cells at the end of the experiment in the colons, spleens, and blood of mice. Data are expressed as mean ± SEM; **p* < 0.05; ***p* < 0.01; *****p* < 0.0001.

***Figure S5 Relative abundances of pro-inflammatory marker in mouse colon after AOM/DSS induction and R.f/F.s administration.***

After the mice were sacrificed, the intestinal tissues were collected and homogenized. And qPCR was performed to detect the relative expression levels of several pro-inflammatory cytokines (IL-1β, IL-2, IL-6, TNF-α and IFN-γ) in mice treated by R.f **(A)** and F.s **(B)**.

**Supplementary Tables**

**Table S1 Antibodies for FACS**

| **Antibody** | **Reagent** | **Manufacturers** |
| --- | --- | --- |
| CD11b | FITC | Biolegand |
| CD3ε | FITC | Biolegand |
| F4/80 | PE | Biolegand |
| CD8α | PE | Biolegand |
| Gr-1 | APC | Biolegand |
| Ly6G | APC | Biolegand |
| CD4 | PE/Cy7 | Biolegand |

**Table S2 Antibodies for WB**

| **Antibody** | **Manufacturers** |
| --- | --- |
| β-Actin | Santa Cruz |
| p65 | Cell Signaling Technology |
| p-p65（S536） | Cell Signaling Technology |

**Table S3 Primers for qPCR**

| **Primer** | **Sequence** |
| --- | --- |
| 16S-F | CCTACGGGAGGCAGCAG |
| 16S-R | ATTACCGCGGCTGCTCTGG |
| R.f-F | CGCGTGGAGGAAGAAGGTTT |
| R.f-R | CACCAGGAATTCCGCTTACCT |
| F.s-F | GGGGAGAAGCATTTCGGTGT |
| F.s-R | TGAATCTTCGCCTACGCTCC |
| E.d-F | AATGCCGCGTGAGTGAAGAA |
| E.d-R | AATGTACCACCTACGCACCC |
